# Thyroid Function in Small for Gestational Age Newborns: A Review

**DOI:** 10.4274/Jcrpe.846

**Published:** 2013-03-01

**Authors:** Franco Bagnoli, Farmeschi Laura, Nappini Sara, Grosso Salvatore

**Affiliations:** 1 Siena University Obstetrics and Reproductive Medicine, Department of Neonatal Pediatrics Section, Siena, Italy; 2 Siena University Obstetrics and Reproductive Medicine Department of Pediatrics, Siena, Italy

**Keywords:** small for gestational age, SGA, thyroid function

## Abstract

Several studies have shown that small for gestational age (SGA) babies have a different hormonal profile than those born with a birth weight appropriate for gestational age (AGA). Thyroid hormones play an important role in growth and neurocognitive development. Only few studies analyzed the concentrations of thyroid-stimulating hormone (TSH) and thyroxine (T4) during fetal and extrauterine life in SGA and AGA newborns, and the existing data on the possible alterations of these hormones in postnatal life are controversial. It remains to be established whether SGA newborns have different blood concentrations of thyroid hormones as compared with AGA infants and if so, whether these findings play a role in the development of obesity, short stature, hypertension, and diabetes - disorders, already known to be related with SGA birth. It has also not yet been established whether and when substitutive therapy with levothyroxine (LT4) should be initiated in preterm and full-term SGA newborns. Further trials are needed to determine the thyroid hormone profile in both preterm and full-term SGA newborns and also to evaluate the effectiveness and safety of LT4 treatment in these infants.

**Conflict of interest:**None declared.

## INTRODUCTION

The term small for gestational age (SGA) newborns has been used to describe a neonate whose weight and/or crown-heel length at birth is at least 2 standard deviations (SD) below the mean for the infant’s GA based on data derived from an appropriate reference population. Some publications define SGA as a birth weight or length below the 3rd, 5th, or 10^th^ percentiles for GA (1,2,3,4). However, -2 SD, a cut-off value which is equivalent to the 2.3^rd^ percentile, is likely to capture the majority of infants with impaired fetal growth. According to recent estimates, 4 115 590 infants were born in the United States in 2004. This means that, at 2.3% of the population, there were approximately 95 000 infants born SGA. 

Intrauterine growth retardation (IUGR) and birth of SGA babies may be related to several factors. These include genetic potential, nutritional status of the mother, placental function and transfer of nutrients, intrauterine hormones and growth factors (5,6,7). There are several publications which show that the restriction of fetal growth may result in long-term development disorders, including delayed growth during childhood, short stature, obesity, and increased risk of hypertension and type 2 diabetes in adulthood (8,9,10,11,12,13,14). Because of these long-term effects, studies on SGA infants may yield intriguing hypotheses about the fetal origins of adult disease (7). Functioning of the hypothalamic-pituitary-adrenal axis may be permanently programmed during development (15). Cianfarani et al (8) reported that children born SGA who do not show catch-up growth have significantly higher fasting levels of plasma cortisol than children born SGA who achieve catch-up growth. 

In addition, cortisol may act by limiting insulin-like growth factor binding protein-3 (IGFBP-3) proteolysis in the perinatal period, thereby minimizing the availability of circulating IGFs and imposing early growth restriction.

Thyroid hormones are crucial for growth and neurocognitive development as well. It remains to be established whether thyroid hormone impairment may play a role in the growth retardation affecting SGA. In fact, very few studies have compared thyroid function in SGA and appropriate for gestational age (AGA) newborns. Conflicting results have been published for neonatal plasma concentrations of thyroxine (T4) and thyroid stimulating hormone (TSH) with diverse study designs including different timing (i.e. in cord blood at birth, in the first days and in the first weeks of life) and different methodology in the evaluation (16,17,18,19,20,21,23,24).

**Thyroid Function in SGA Infants **

**Experimental Studies**

Only a few studies on animal models have been reported in the literature on SGA fetuses. When compared to normal controls, SGA newborn lambs show overlapping TSH and reverse triiodothyronine (rT3) plasma levels. By contrast, plasma T3, total T4 (TT4) and free T4 (FT4) concentrations are significantly lower. Thyroid size, expressed as relative to body weight, is higher in SGA newborns, suggesting that a partial compensation for low thyroid hormone levels may occur during fetal life. Plasma TSH and T4 concentrations show a similar increase after exposure to cold and thyrotropin-releasing hormone (TRH) or TSH administration in SGA and control lambs; however, in the former, the rise in T3 levels is constantly depressed in all stimulation tests.

**Fetal Thyroid Function**

Cordocentesis has permitted the study of fetal thyroid function in utero. In normal fetuses, concentrations of TSH, thyroxine-binding globulin (TBG), and thyroid hormones increase progressively during intrauterine life. Fetal TSH concentrations are always higher compared to nonpregnant adult values. Physiologically, TBG concentrations reach adult levels at the end of gestation. While TT4 and FT4 concentrations reach adult levels by 36 weeks of gestation, TT3 and FT3 remain always lower than adult levels. There are no significant associations between fetal and maternal concentrations of TRH, TBG, or thyroid hormones. Maternal administration of TRH from at least 25 weeks gestation stimulates the fetal pituitary gland to produce TSH. The response is rapid, unrelated to gestational age, and much greater than that of the mother. These findings suggest that in intrauterine life, maturation of the pituitary, thyroid, and liver are independent and autonomous. 

Thyroid function has been evaluated in fetal blood samples obtained by funipuncture (cordocentesis) from SGA fetuses between 20-21 and 38-39 weeks' gestation (19,20,21,22,23,24,25,26) (Table 1). When compared to AGA fetuses, T4 and FT4 levels were found to be significantly lower in SGA fetuses (19,26). No differences seem to be present in the T3 and FT3 levels (19). With respect to TSH levels in SGA, controversies still remain since they have been found significantly elevated in some studies but not in others. According to Thorpe-Beeston et al (19), the high TSH and the low FT4 concentrations are significantly associated with the severity of fetal hypoxemia and acidemia. It has been pointed out that the low serum levels of thyroid hormones may have some beneficial effects in SGA population by reducing oxygen requirements, even though they may adversely affect the brain development (25). 

Although circulating concentrations of FT4 are major determinants of cellular uptake of T4 and T3, other factors such as the expression and function of thyroid hormone receptors (TRs) may modulate thyroid hormone action at the tissue level. Ligand binding studies have shown high affinity nuclear binding sites for T3 in the human fetal brain (28) and in the placenta (29). In the latter tissue, T3 may stimulate the production of 17β estradiol and epidermal growth factor (30) suggesting a T3 role in the control of trophoblast growth and development, which are both abnormal in IUGR (31).

Placental thyroid receptor α1, α2 and β1 expression was shown to be significantly upregulated in pregnancies complicated by IUGR when compared to normal pregnancies (27). Although it remains to be established whether the reductions in circulating thyroid hormones determine hypothyroidism at the tissue level, the increased expression of TRs may support the hypothesis of a maintenance of tissue euthyroidism in the face of reduced circulating concentrations of thyroid hormones (27).

**Thyroid Function at Birth**

Significant changes of thyroid function occur at birth in the term newborn. A surge in the serum TSH is seen at 30 minutes after delivery (up to 60-70 mU/L). The increasing TSH serum levels are thought to be related to the cold exposure in the ambient atmosphere. The high TSH peak concentration stimulates production of both T4 and T3 in the thyroid gland. In the first postnatal week, the T4 serum levels reach concentrations that are higher than at any other time of life. The T3 serum levels continue to rise during the first 28 days. This is the consequence of both the direct TSH stimulation and the increased postnatal expression of the activating deiodinase D1 and the loss of placental D3. Thyroid hormone profile in SGA babies at birth remains to be clearly defined (Table 1). Early studies (32) showed that TSH levels in cord blood samples were not significantly higher in AGA babies as compared to SGA ones, both in full-term and preterm newborns. Analogously, higher cord blood T4 levels were not observed in AGA as compared to SGA babies. These findings have been confirmed by recent studies (22,24). In particular, Rashmi et al (22) found that cord blood TSH levels were not influenced by IUGR and gender, while they were negatively correlated with GA and birth weight and tended to be increased by perinatal stress factors such as birth asphyxia and difficult deliveries (22). By contrast, Nieto-Diaz et al (23) found that SGA babies at birth have significantly lower cord blood TSH and IGF-I levels associated with higher growth hormone levels. This endocrine profile is similar to that observed in malnourished children suggesting similar pathophysiological mechanisms for both IUGR and post-natal growth retardation of nutritional etiology (33). Significantly higher cord blood TSH levels and lower T4 concentrations were observed by Setia et al (20) who also noted a significant positive insulin sensitivity with TSH and a negative association with T4 levels. This means that thyroid hormones may play a key role in the increased insulin sensitivity at birth in IUGR. Several researchers argue against a pathogenetic role of placental deiodinases in hypothyroxinemia observed in IUGR fetuses, as no difference in deiodinase expression has been detected in normal and IUGR placentas (34). A possible pathogenetic role may be played by the transthyretin (TTR), a placental high-affinity TBG, which is involved in maternal thyroid hormone uptake by the fetus by modifying deiodination. In that perspective, alteration in thyroid hormone profile and insulin sensitivity may be the epiphenomena of growth retardation. In fact, an impairment in the TTR production or secretion occurs in IUGR with reduced maternal transfer of thyroid hormones to the fetus which accounts for circulating hypothyroxinemia (20). 

**Thyroid Function in the Early Neonatal Period**

Very few studies evaluated thyroid hormone profile in SGA babies during the first days of life (Table 1). Early reports demonstrated that SGA newborns have TSH basal levels significantly higher than preterm AGA babies. By contrast, no differences have been found in T4 and T3 serum levels or in pituitary-thyroid responsiveness to TRH stimulation. Jacobsen et al (21) showed that the percentage increase in serum TSH after intravenous injection of 40 μg TRH, between 5 and 167 hours after birth, is overlapping in SGA and AGA preterm babies and similar to that observed in full-term newborns. Interestingly, although higher than those observed in preterm neonates, serum T4 levels in SGA persist at significantly lower levels than in full-term AGA newborns up to the 49^th^ day of life when, according to Jacobsen et al (21), serum T4 levels reach normal concentrations. 

**Figure 1:**
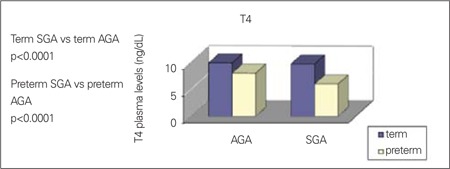
Plasma concentration of thyroxine (T4) in term and pretermappropriate for gestational age (AGA) and small for gestational age (SGA)newborns; Adapted from Bagnoli et al (35)

**Figure 2:**
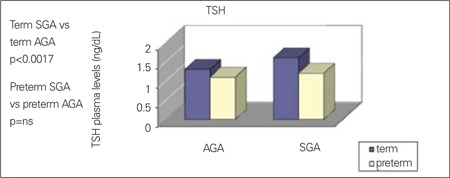
Plasma concentration of thyroid stimulating hormone (TSH) in termand preterm appropriate for gestational age (AGA) and small for gestationalage (SGA) newborns; Adapted from Bagnoli et al (35)

We have recently evaluated the relationship between growth restriction and thyroid function in the first week of life using data from screening for congenital hypothyroidism. Plasma concentrations of TSH and T4 have been analyzed in 14 092 newborns (13 333 AGA and 759 SGA infants) (35). We observed that preterm and full-term SGA infants had lower T4 serum levels, whereas concentrations of TSH were significantly higher only in full-term SGA infants (Figure 1, 2). Moreover, T4 levels were positively correlated with GA in SGA and AGA groups, whereas TSH concentrations were correlated with GA only in the AGA group. SGA babies were affected by a significantly higher recall rate for TSH and T4 anomalies than AGA newborns (1.84% vs. 0.93%). However, in most cases, these alterations reverted to normal during the following months. In this context, it should be underlined that in our study, all babies who were subsequently diagnosed as affected by hypothyroidism belonged to the AGA group (35). Therefore, hypothyroxinemia found in SGA newborns is commonly transient and tends to disappear by the next follow-up. The spontaneous normalization of the blood T4 level in SGA babies may be interpreted as the recovery of thyroid function as the consequence of adequate nutrition. Of course, a more accurate monitoring of the thyroid function in this population may be necessary. A persistent reduced secretion of T4 during the first days of life in SGA babies may be due to pathogenetic mechanisms similar to those hypothesized in IUGR newborns, in which the low T4 plasma levels have been related to retarded development of the gland caused by malnutrition and by placental hypoxia. Indeed, SGA newborns are typically undernourished babies (36,37). However, we also confirmed that SGA newborns show higher TSH serum levels than AGA controls. This finding may be interpreted as a correct pituitary response to the low T4 levels observed in SGA population. Therefore, it is possible that the pituitary gland function might not be influenced by nutritional status. The appropriate pituitary functioning and the lack of any relationship with malnutrition in SGA newborns seem to be further supported by investigations on the adrenocorticotropic hormone (ACTH)-adrenal axis, demonstrating higher ACTH levels and lower cortisol concentrations than in AGA babies (38). 

Finally, malnutrition may affect thyroid hormone synthesis through reduced availability of phenylalanine and tyrosine leading to low plasma concentrations of T4 in utero and in the first days of life. This endocrine and metabolic state could be an advantage in conditions of poor nutrition or pathologies leading to growth retardation, because it is associated with reduced oxygen consumption. 

**Management**

Preterm babies show lower plasma thyroid levels and are at higher risk for hypothyroxinemia than newborns at term. On that basis, some authors pointed out that substitutive therapy with levothyroxine (LT4) might be useful in these infants. However, evidence supporting the effectiveness of LT4 therapy in this group of infants is lacking and no agreement exists about LT4 doses or duration of therapy. According to our observations, full-term SGA babies show significantly lower thyroid plasma levels than full-term AGA newborns. In addition, we demonstrated that preterm SGA babies have even significantly lower thyroid plasma levels when compared to preterm AGA newborns. 

Since many authors suggested that LT4 prophylaxis may well be appropriate in preterm babies less that 27 weeks’ gestation, we believe that a substitutive therapy with LT4 may represent a rational strategy also in preterm SGA newborns. However, the time at which LT4 prophylaxis should be started is a main issue which needs to be addressed. It is currently suggested that when low plasma FT4 levels are observed, preterm babies should be given LT4 prophylaxis between the 1st and the 2^nd^ months of life. Considering the cardinal role played by thyroid hormones on central nervous system and lung development, in our clinical practice, FT4 and TSH levels are evaluated between the 15^th^ and 20^t^h day after birth (17,18). When FT4 plasma levels are found at the lower normal ranges, preterm SGA babies are placed under substitutive LT4 therapy (2 μg/kg/day). Evaluation of FT4 levels is commonly scheduled for every 8-10 days of therapy and when necessary, LT4 doses are readjusted. 

As already mentioned, full-term SGA newborns show significantly lower thyroid levels than full-term AGA babies. However, no studies addressing the question of LT4 prophylaxis in this population have been reported. On the other hand, there are studies showing that TSH levels in the upper normal range (>2 mIU/L) might be correlated with increased risk of developing hypothyroidism and cardiovascular disorders later in life (8,39,40,41,42). Moreover, there are several studies pointing to the high risk of SGA newborns for developing endocrine-metabolic disorders later in life (8,9,10,11,12,13,14).

On the basis of these studies, neonatologists should consider the potential role of LT4 prophylaxis (low doses) in full-term SGA babies. Awaiting appropriate studies on that question, an adequate monitoring of thyroid function may be useful in term SGA babies. Indeed, case-control studies are needed to evaluate the potential effectiveness and usefulness of LT4 treatment in both preterm and full-term SGA newborns. 

## Figures and Tables

**Table 1:**
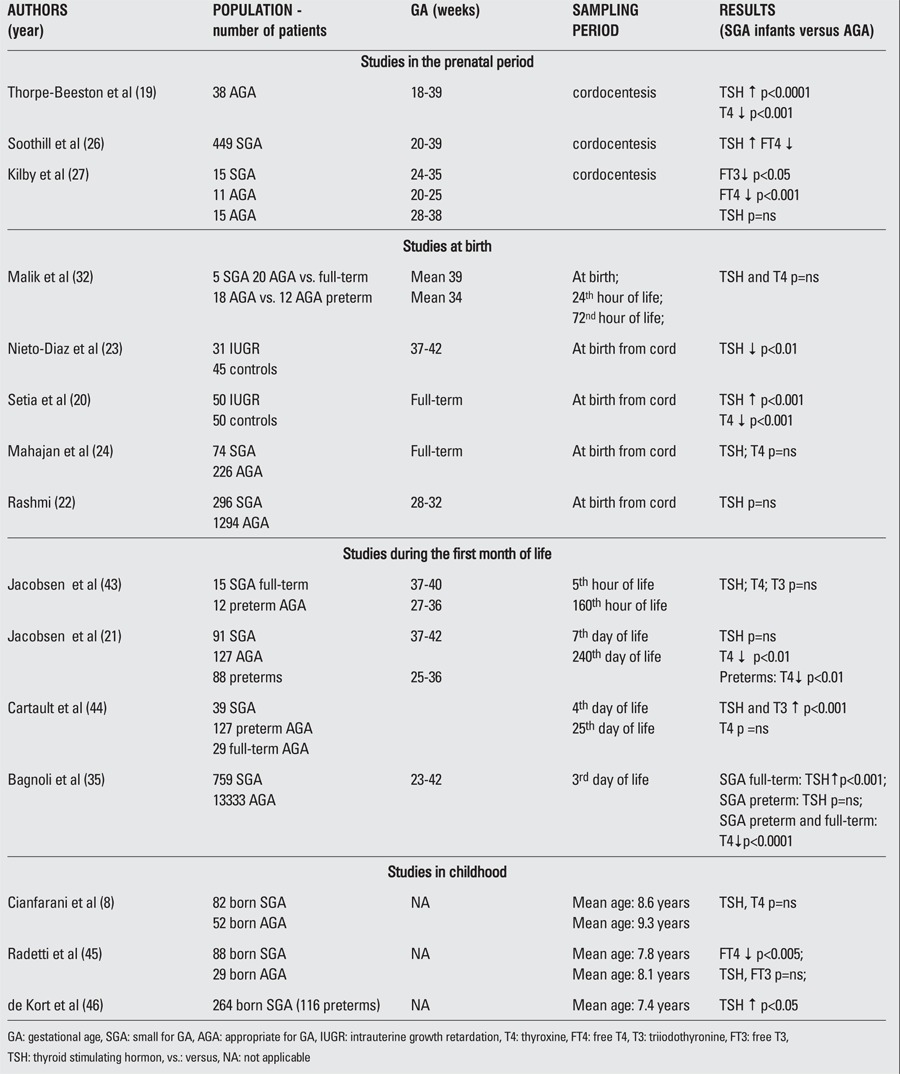
Published studies on thyroid hormones in SGA fetuses, newborns and children
